# TBX21 predicts prognosis of patients and drives cancer stem cell maintenance via the TBX21–IL-4 pathway in lung adenocarcinoma

**DOI:** 10.1186/s13287-018-0820-6

**Published:** 2018-04-03

**Authors:** Shuangtao Zhao, Wenzhi Shen, Jiangyong Yu, Luhua Wang

**Affiliations:** 1 Department of Radiation Oncology, National Cancer Center/Cancer Hospital, Chinese Academy of Medical Sciences and Peking Union Medical College, Beijing, 100021 China; 2 Department of Pathology and Institute of Precision Medicine, Jining Medical University, Jining, 272067 China; 3 The School of Medicine, Nankai University, Tianjin, 300071 China

**Keywords:** Lung adenocarcinoma, TBX21, Prognosis, Cancer stemness, IL-4

## Abstract

**Background:**

The Th1 cell-specific transcription factor TBX21 functions as a regulator of expression of a Th1 cytokine, interferon gamma (IFN-γ). However, the specific function of TBX21 correlated with cancer stemness remains unclear.

**Methods:**

Using univariate and multivariate survival analysis, TBX21was identified as an independent predictive factor and was associated with poor prognosis in 1389 patients with lung adenocarcinoma (LUAD). Its mechanism in the prognosis was explored by functional enrichment analysis and validated in bioexperiments.

**Results:**

In the training and test sets, TBX21 could classify 1389 LUAD patients into high and low-risk groups with significantly different prognosis (*P* < 0.01). Its prognostic power was independent of other clinical factors including stage, age, gender and smoking status. Functional studies indicated that downregulating TBX21 in lung cancer cells decreased the fraction of cancer stem cells and their sphere and tumor initiation frequency. Furthermore, the study showed that TBX21 activation transduced a TBX21–IL-4 signaling cascade to promote tumor initiation, tumor growth and expression of stemness markers.

**Conclusions:**

These data demonstrated a key role of TBX21 in the maintenance of cancer stemness and that the TBX21–IL-4 pathway is a crucial factor contributing to lung carcinogenesis.

**Graphical abstract:**

TBX21 prognostic model correlated with cancer stemness via TBX21-IL-4 pathway in LUAD patients
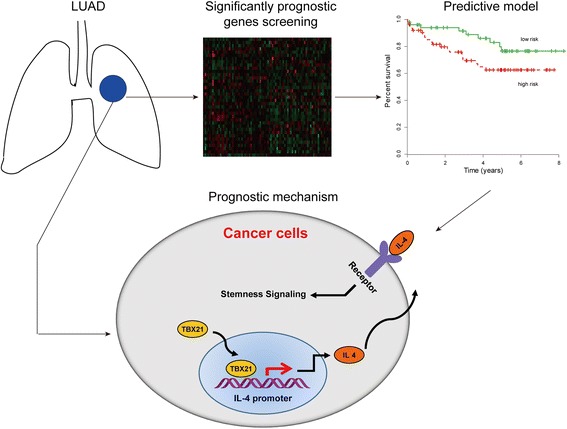

**Electronic supplementary material:**

The online version of this article (10.1186/s13287-018-0820-6) contains supplementary material, which is available to authorized users.

## Background

Lung cancer remains the most common cancer in the last decades and is still the primary cause of cancer death worldwide, including China [1]. Nonsmall cell lung cancer (NSCLC) accounts for approximately 85% of lung cancers [2] and lung adenocarcinoma (LUAD) is currently the predominant histological subtype of NSCLC. Although recent advances in multimodality therapy improve the clinical outcome [3], patients with LUAD still have a high rate of relapse (30–66%) [4, 5] and will die because of cancer recurrence [5, 6]. Despite morphological assessment for stratifying patients into different risk groups, it is clear that the overall survival (OS) rates were still 13–58.3% at 5 years for LUAD patients with high invasiveness and early metastasis [2]. Therefore, it is urgent to develop novel molecular prognosis biomarkers for predicting the prognosis and identifying the high-risk subgroup of LUAD patients with early stage who might benefit from comprehensive therapy.

Previous studies reported that stemness of cancer stem cells (CSCs) may enable CSCs to metastasize and regrow tumors [7], and can be acquired by nonstem cancer cells as they dedifferentiate in response to multiple stimuli [8, 9] including inflammatory response. The inflammatory microenvironment in the tumor is activated to drive tumor development as the seventh hallmark of cancer [8]. Li et al. [10] reported that the inflammation-related gene *IL-1β* promotes stemness and invasiveness of colon tumor through activation of CSC self-renewal and EMT. Zhao et al. [11] reported that a three inflammatory gene model including *IL-6*, *IL-1A* and *CSF3* could predict survival of diffuse large B-cell lymphoma patients, and patients with high-risk score of this signature had significantly shorter survival than those with low-risk score. The pathway analysis comprised of IRGs could determine genetic risk factors for cancers that might have an underappreciated modest inflammatory component.

TBX21 is a member of a phylogenetically conserved family of genes and Tbx21 protein is a Th1 cell-specific transcription factor that controls expression of the hallmark Th1 cytokine, interferon gamma (IFN-γ) [12, 13]. Recently, an increased incidence of TBX21 has been linked to cancer development [14, 15]. However, the specific function of TBX21 associated with cancer stemness or the details remains unclear. In this study, to develop a reliable prognostic model identifying LUAD patients with high-risk status, we screened the significant gene TBX21 from 1027 IRGs, constructed a model to predict survival and further validated it in GEO datasets. Then we investigated the potential biological function of TBX21 in disease progression with biological experiment and confirmed that TBX21 promoted the cancer stemness of tumor cells to reduce the survival time of patients with LUAD.

## Methods

### Patient datasets

The clinical data and gene expression profiles of LUAD patients and normal controls were downloaded from the GEO datasets (https://www.ncbi.nlm.nih.gov/gds) and The Cancer Genome Atlas (TCGA) (https://cancergenome.nih.gov). After removal of LUAD patients with missing values, a total of 1421 LUAD patients and 51 normal controls were analyzed in this study, including 116 patients form GSE50081, 159 patients from GSE31210, 71 patients from GSE30219, 302 patients from GSE72094, 399 patients from TCGA, 342 patients and 19 normal controls from GSE68465, and 32 patients and 32 normal controls from GSE32863. Detailed clinical features of patients from the training data and validated data are presented in Table 1.

**Table 1:** Correlation between TBX21 expression and different clinical characteristics in LUAD patients enrolled in the study

Variable	Low TBX21 expression group	HighTBX21 expression group	*P* value^#^
GSE50081			
Stage			0.385
I	37 (78.7%)	45 (71.4%)	
≥ II	10 (21.3%)	18 (28.6%)	
Age			0.245
< 65 years	14 (26.4%)	23 (36.5%)	
≥ 65 years	39 (73.6%)	40 (63.5%)	
Gender			0.877
Female	26 (49.1%)	30 (47.6%)	
Male	27 (50.9%)	33 (52.4%)	
Smoking			0.364
No	11 (20.8%)	9 (14.3%)	
Yes	35 (66.0%)	49 (77.8%)	
NA	7 (13.2%)	5 (7.9%)	
GSE31210			
Stage			0.296
I	74 (78.7%)	45 (71.4%)	
≥ II	20 (21.3%)	18 (28.6%)	
Age			0.107
< 65 years	71 (74.0%)	39 (61.9%)	
≥ 65 years	25 (26.0%)	24 (38.1%)	
Gender			0.382
Female	51 (53.1%)	29 (46.0%)	
Male	45 (46.9%)	34 (54.0%)	
Smoking			0.419
No	52 (54.2%)	30 (47.6%)	
Yes	44 (45.8%)	33 (52.4%)	
GSE30219			
Stage			0.801
I	27 (87.1%)	34 (85%)	
≥ II	4 (12.9%)	6 (15%)	
Age			0.583
< 65 years	19 (73.1%)	26 (66.7%)	
≥ 65 years	7 (26.9%)	13 (33.3%)	
Gender			0.376
Female	9 (29.0%)	8 (20.0%)	
Male	22 (71.0%)	32 (80.0%)	
GSE68465			
Stage			0.448
I	62 (36.3%)	55 (32.4%)	
≥ II	109 (63.7%)	115 (67.6%)	
Age			0.746
< 65 years	85 (49.7%)	82 (48.0%)	
≥ 65 years	86 (50.3%)	89 (52.0%)	
Gender			0.175
Female	81 (47.4%)	93 (54.7%)	
Male	90 (52.6%)	77 (45.3%)	
Smoking			0.105
No	19 (11.1%)	22 (12.9%)	
Yes	108 (63.2%)	121 (70.8%)	
NA	44 (25.7%)	28 (16.3%)	
GSE72094			
Stage			0.570
I	99 (63.5%)	88 (66.7%)	
≥ II	57 (36.5%)	44 (33.3%)	
Age			0.207
< 65 years	51 (30.0%)	31 (23.5%)	
≥ 65 years	119 (70.0%)	101 (76.5%)	
Gender			0.955
Female	92 (54.1%)	71 (53.8%)	
Male	78 (45.9%)	61 (46.2%)	
Smoking			0.103
No	21 (12.4%)	7 (5.3%)	
Yes	122 (71.8%)	100 (75.8%)	
NA	27 (15.8%)	25 (18.9%)	
TCGA LUAD			
Stage			0.805
I	114 (59.1%)	118 (57.8%)	
≥ II	79 (40.9%)	86 (42.2%)	
Age			0.022*
< 65 years	95 (47.5%)	7 (5.3%)	
≥ 65 years	98 (49.0%)	100 (75.8%)	
NA	27 (15.8%)	25 (18.9%)	
Gender			0.337
Female	104 (52.0%)	113 (56.8%)	
Male	96 (48.0%)	86 (43.2%)	
Smoking			0.056
No	71 (35.5%)	53 (26.6%)	
Yes	129 (64.5%)	146 (73.4%)	

*LUAD* lung adenocarcinoma, *TCGA* The Cancer Genome Atlas, *NA* not available

^#^Pearson’s chi-square test

*Fisher’s exact test

### Statistical analysis

Cutoff values were established based on the median expression of the associated genes in each dataset. Pearson’s chi-square test analysis of variance was used to analyze statistical significance in demographic and clinical characteristics [16]. The *t* test was used to compare the distributive difference of the associated gene expression between patients and normal controls, or between the high-risk score group and the low-risk score group. The correlation between TBX21 and IL-4 expression was calculated with Spearman correlation analysis in the integrated GEO dataset (*n* = 1389). The association between TBX21 expression value and OS of patients was evaluated by univariate Cox proportional hazards regression analysis. Survival differences between low and high-risk groups in each dataset were assessed by Kaplan–Meier estimation, and compared by log-rank test [17]. Multivariate Cox proportional hazards regression analysis and data stratification analysis were performed in our study to explore whether the predictive power of TBX21 was independent of the other clinical factors. Hazard ratios (HRs) and 95% confidence intervals (CIs) were calculated in each dataset. The prognostic performance at 3, 5 or 10 years was measured by time-dependent ROC curves. Statistical analysis was conducted with SPSS 13.0, and presented with R3.2.5 software. Results were considered statistically significant at *P* < 0.05.

### Functional enrichment analysis

DAVID Bioinformatics Tool was utilized to perform the functional enrichment of target genes. The biological processes associated with protein-coding genes were identified by conducting gene ontology analysis with this tool. In our study, *P* < 0.05 was defined as a significant threshold. The pathways involved in the targeted gene were predicted by functional enrichment analysis of KEGG in DAVID Bioinformatics Resources 6.8 (https://david.ncifcrf.gov). Finally, the Enrichment Map plugin for Cytoscape was applied to visualize the biological process organization.

### Cell culture

Human lung cancer cell line A549 was purchased from ATCC and was recently authenticated by cellular morphology and short tandem repeat analysis at Microread Inc. (Beijing, China; May 2014) according to the guideline from ATCC. A549 cells were infected with lentivirus carrying pLV-H1-shRNA-puro or pLV-EF1α-TBX21-puro plasmid, followed by selection using 2 μg/ml puromycin to generate polyclonal cell populations.

### Immunohistochemistry

Immunostaining was performed on paraffin human lung cancer tissue slices. Expression levels of TBX21 in the slices were scored according to the percentage of TBX21-positive cells in each lung tissue. The images were recorded by Olympus BX51 Epi-fluorescent microscopy under a 10× or 40× objective (Olympus Co., Tokyo, Japan) [18].

### Western blotting

Cell lysates from different cell lines were prepared with RIPA buffer in the presence of protease inhibitor cocktails and Phosphatase Inhibitor Cocktail 2 and 3 (P8340, P5726 and P0044; Sigma-Aldrich, St Louis, MO, USA). Protein (20–50 μg) was separated by 8–15% Tris–acrylamide gels and transferred onto PVDF membrane The membrane was blocked in 5% skim milk, subsequently incubated with primary antibodies at 4 °C overnight followed by incubation with peroxidase-conjugated goat anti-mouse IgG or goat anti-rabbit IgG and developed with Pierce ECL reagent (catalog #17153; Millipore, Billerica, MA, USA) [19].

### Flow cytometry

We stained 1 × 10^6^ tumor cells/ml with either 5 μg/ml HOECHST 33342 dye or HOECHST dye plus 100 mM Verapamil hydrochloride, to block dye efflux, at 37 °C for 30 min, as described previously (http://www.bdbiosciences.com). At the end of the staining period, cells were resuspended in cold staining buffer containing 2 μg/ml propidium iodide (PI) for dead cell discrimination. The HOECHST dye was excited at 350 nm, and its fluorescence measured at two wavelengths (450/20 nm band-pass filter and 675LP optical filter).

### Mammosphere formation assay

Cells were collected and rinsed to remove serum, then dissociated to single-cell suspension in serum-free DMEM/F12 medium supplemented with 100 IU/ml penicillin, 100 μg/ml streptomycin, 20 ng/ml human recombinant epidermal growth factor (hREGF), 20 ng/ml human recombinant basic fibroblast growth factor (bFGF) and 2% B27 supplement (Invitrogen). Cells were subsequently cultured in ultralow-attachment 24-well plates (Corning Inc.) at a density of no more than 500 cells/well.

### Luciferase assay

To construct the luciferase reporter vectors, the IL-4 gene promoter fragment was amplified from human genomic DNA and cloned into the firefly luciferase plasmid pGL3-basic-IRES. For reporter assays, the aforementioned constructs along with pRL-TK plasmid (internal reference) were cotransfected into A549 cells. Cell lysates were then collected and analyzed with the Dual-Luciferase Reporter Assay System (E1910; Promega) following its manual.

### Chromatin immunoprecipitation assays

Chromatin immunoprecipitation (ChIP) assays were performed as described previously (17-371EZ-CHIP, Millipore, Billerica, MA, USA). Briefly, after sonication, 10% of each sample was saved as total input, 45% of each sample was incubated with 5 μg of anti-TBX21 antibody (catalog #sc-126; Santa Cruz, TX, USA), and the remaining 45% of each sample was incubated with 5 μg of normal IgG (catalog #sc-2025; Santa Cruz) at 4 °C overnight and then with 50 μl (bead volume) of Pure Proteome proteinG magnetic beads (catalog #LSKMAGG02; Millipore, CA, USA) at 4 °C for 4 h. After washing and reverse crosslinking, DNA was extracted with phenol–chloroform, precipitated with ethanol and dissolved in water. The TBX21 binding sites in the IL-4 gene promoter region were identified by Chip-PCR.

### Animal study

A549-shTBX21 and A549-sc cells (5 × 10^6^ cells) were injected into NOD/SCID mice subcutaneously and tumors were palpable at 12 days after injection. Eighteen days after injection the mice were sacrificed, and tumors were harvested and analyzed. The tumors were then digested with enzyme into single cell suspension, and cell sorting was performed to sort out the side population cells using FACS. Further, the mRNA and proteins were extracted from the sorted SP cells.

## Results

### Upregulation of TBX21 was associated with poor prognosis of patients with lung adenocarcinoma

To investigate IRGs’ prognostic role in LUAD, 1027 IRGs (Additional file 1: Table S1) from others’ previous research [20] were firstly evaluated in 1389 LUAD patients from six datasets. With the median value as a cutoff point, upregulation of TBX21 was identified as a poor prognostic biomarker in patients with LUAD. The clinical baseline characteristics were not significantly different between the low and high-expression groups of TBX21 (Table 1).

**Fig. 1:**
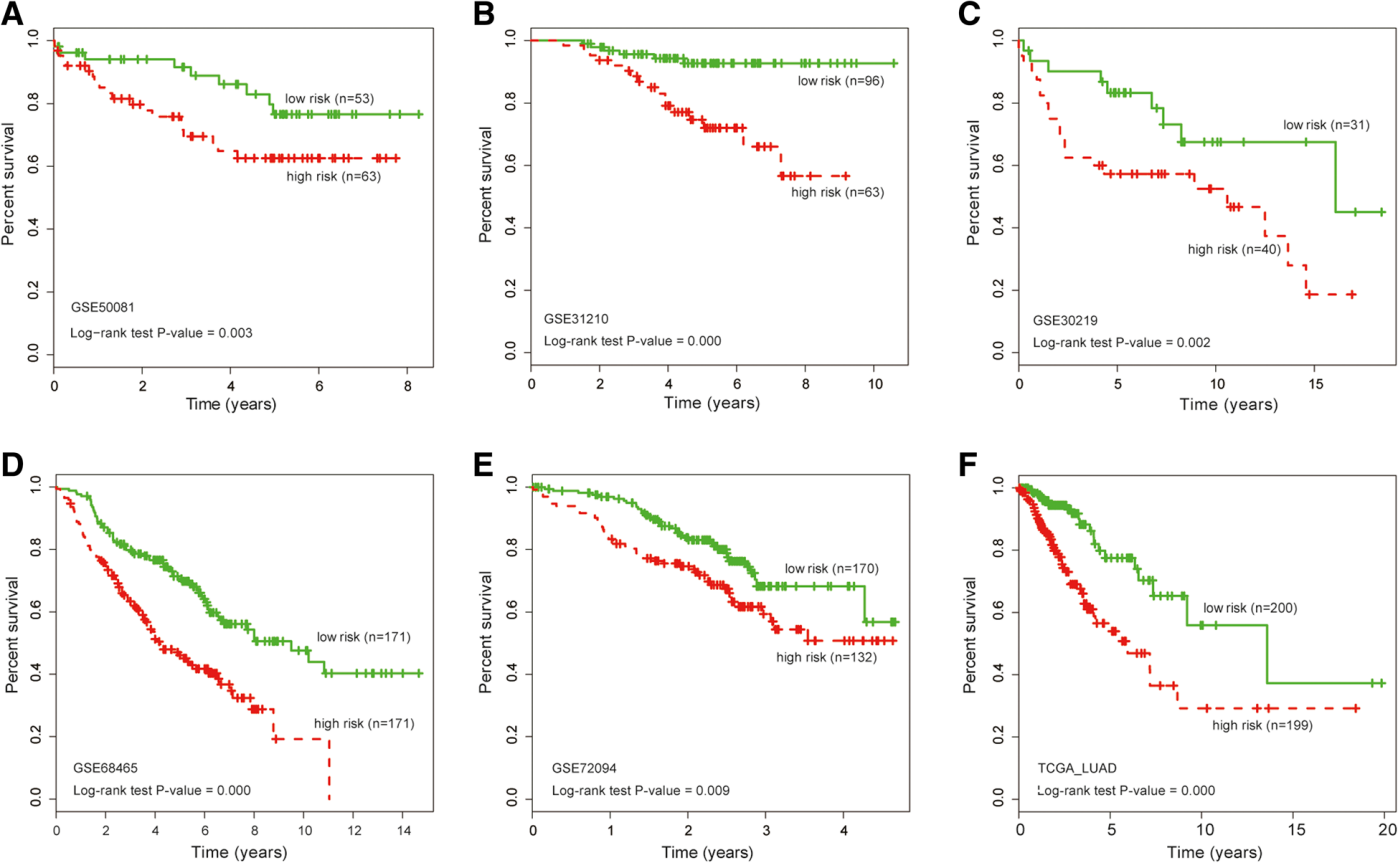
Prognostic model validating predictive power of TBX21 gene in independent datasets. Kaplan–Meier survival curves between low-risk group and high-risk group in (**a**) training dataset and (**b**–**f**) other validated datasets

Next, the log-rank test was accomplished between the low and high-value groups in this dataset. We found that patients with high-value expression of TBX21 had significantly higher risk than those with low values (HR = 2.008, 95% CI 1.284–3.396, *P* = 0.003; Fig. 1a). The 3-year OS rates and the 5-year OS rates of patients in the high-risk group were 69.53% and 62.58%, respectively, whereas the corresponding rates in the low-risk group were 93.30% and 78.09%. These results indicated that TBX21 could distinguish LUAD patients with high or low risk of survival.

To assess the reproducibility of the prognostic model, another 1273 patients from five independent datasets (accession numbers GSE31210, GSE30219, GSE68465, GSE72094 and TCGA LUAD) were used to validate its predictive ability. With the same cutoff value derived from the training dataset, LUAD patients in each dataset were then divided into two groups: high and low-risk groups. Similar to the earlier result, the significantly different survival between the two predictive groups (*P* < 0.01; Fig. 1b–f) was demonstrated by survival analysis. In GSE31210, the 3-year OS rates and the 5-year OS rates of patients in the high-risk group were less than the corresponding rates in the low-risk group (88.57% and 74.70% vs 95.59% and 92.71%), respectively (Fig. 1b). The LUAD patients with high-risk scores showed significantly shorter survival than those with low-risk scores (median survival 10.60 years vs 16.10 years (*P* = 0.030) in GSE30219, 4.18 years vs 9.50 years (*P* = 2.58 × 10^–6^) in GSE68465, 3.07 years vs 4.27 years (*P* = 0.012) in GSE72094, and 5.95 years vs 13.58 years (*P* = 8.03 × 10^–6^) in TCGA LUAD) (Fig. 1c–f). These results demonstrated that the prognostic model of TBX21 was robust to identify LUAD patients with poor survival.

### The prognostic power of TBX21 was independent of other clinicopathological factors

To evaluate whether the prognostic power of TBX21 was independent of other clinical factors, multivariate Cox proportional hazard regression analysis was performed with risk score and other available clinicopathological variables (including patients’ age, gender, clinical stage and smoking status) as covariates in these six LUAD datasets (Table 2). The results showed that TBX21 was still significantly correlated with survival when adjusted for age, gender, clinical stage and smoking status in the training dataset (HR = 1.943, 95% CI 1.234–3.060, *P* = 0.004) and in the other five validated datasets (HR = 1.018, 95% CI 1.002–1.035, *P* = 0.028 for GSE31210; HR = 2.625, 95% CI 1.284–5.365, *P* = 0.008 for GSE30219; HR = 1.009, 95% CI 1.007–1.012, *P* = 0.000 for GSE68465; HR = 1.469, 95% CI 1.127–1.914, *P* = 0.004 for GSE72094; and HR = 1.059, 95% CI 1.008–1.112, *P* = 0.023 for TCGA LUAD; Table 2). However, we also discovered that clinical stage, age and gender were significant in the multivariate analysis in some datasets besides the predictive gene TBX21.

**Table 2:** Univariate and multivariate Cox regression analysis for lung adenocarcinoma in validated datasets

Variable	Univariate analysis			Multivariate analysis		
	HR	95% CI	*P* value	HR	95% CI	*P* value
GSE31210 (*n* = 159)						
Gender (male/female)	1.484	0.651–3.387	0.348			
Stage (≥ II/I)	3.107	1.358–7.110	0.007	2.971	1.295–6.819	0.010
Age (≥ 65/< 65 years)	1.854	0.813–4.231	0.142			
Smoking (yes/No)	2.101	0.907–4.867	0.083			
TBX21 (high risk/low risk)	4.550	1.960–10.560	0.000	1.018	1.002–1.035	0.028
GSE30219 (*n* = 71)						
Gender (male/female)	1.111	0.464–2.661	0.813			
Stage (≥ II/I)	1.669	0.878–3.173	0.118			
Age (≥ 65/< 65 years)	2.539	1.250–5.158	0.010	2.267	1.108–4.637	0.025
TBX21 (high risk/low risk)	2.876	1.457–5.677	0.002	2.625	1.284–5.365	0.008
GSE68465 (*n* = 342)						
Gender (male/female)	1.376	1.008–1.878	0.044	1.425	1.033–1.965	0.031
Stage (≥ II/I)	1.713	1.362–2.153	0.000	1.621	1.272–2.066	0.000
Age (≥ 65/< 65 years)	1.266	0.928–1.726	0.137			
Smoking (yes/no)	1.045	0.626–1.744	0.866			
TBX21 (high risk/low risk)	2.147	1.561–2.952	0.000	1.009	1.007–1.012	0.000
GSE72094 (*n* = 302)						
Gender (male/female)	1.500	0.976–2.305	0.065			
Stage (≥ II/I)	2.593	1.676–4.011	0.000	2.682	1.734–4.149	0.000
Age (≥ 65/< 65 years)	1.485	0.882–2.501	0.137			
Smoking (yes/no)	1.966	0.714–5.413	0.191			
TBX21 (high risk/low risk)	1.424	1.091–1.859	0.009	1.469	1.127–1.914	0.004
TCGA LUAD (*n* = 399)						
Gender (male/female)	0.943	0.601–1.480	0.798			
Stage (≥ II/I)	3.079	1.920–4.937	0.000	2.956	1.838–4.754	0.000
Age (≥ 65/< 65 years)	1.375	0.865–2.184	0.178			
Smoking (yes/no)	1.102	0.676–1.796	0.698			
TBX21 (high risk/low risk)	2.793	1.779–4.384	0.000	1.059	1.008–1.112	0.023

*HR* hazard ratio, *CI* confidence interval, *LUAD* lung adenocarcinoma, *TCGA* The Cancer Genome Atlas

Next, data stratification analysis was carried out according to clinical stage, age or gender in the integrated GEO datasets. First, all patients from the integrated GEO datasets were stratified into two groups with different stage status (stage I, *n* = 797 and stage ≥ II, *n* = 566). As shown in Additional file 2: Figure S1A, the cutoff value of TBX21 could subdivide patients with stage I into the high-risk group and low-risk group with significant survival time (HR = 2.876, 95% CI 2.087–3.963, *P* = 7.02 × 10^–11^). The OS rates of patients with high-risk score were 76.93% and 65.70% at 3 and 5 years, respectively, which were also significantly lower than those from patients with low-risk score whose corresponding proportions were 93.09% and 84.94%. For the patients with stage ≥ II, we obtained a similar result to patients with stage I (HR = 2.170, 95% CI 1.663–2.832, *P* = 9.24 × 10^–8^; Additional file 2: Figure S1B). Analogously, the predictive power of TBX21 was further tested for patients with different age or gender. The patients of each subgroup were subclassified into two groups (high and low-risk groups) with significantly different clinical outcome (*P* < 0.001). The OS rates of patients with high-risk score were significantly worse than those with low-risk score (at 3 and 5 years, 73.12% vs 87.23% and 59.12% vs 79.60% in younger group (Additional file 2: Figure S1C), 72.42% vs 86.56% and 58.41% vs 76.36% in female group (Additional file 2: Figure S1E); median survival (years) 5.04 vs 10.20 (*P* < 0.0001, Additional file 2: Figure S1D) in older group and 5.55 vs 10.83 (*P* < 0.0001, Additional file 2: Figure S1F) in male group). Generally, these results demonstrated that the TBX21 predictive power for prognosis was independent of other clinicopathological factors for patients with LUAD.

### Validation of high expression of TBX21 in LUAD

**Fig. 2:**
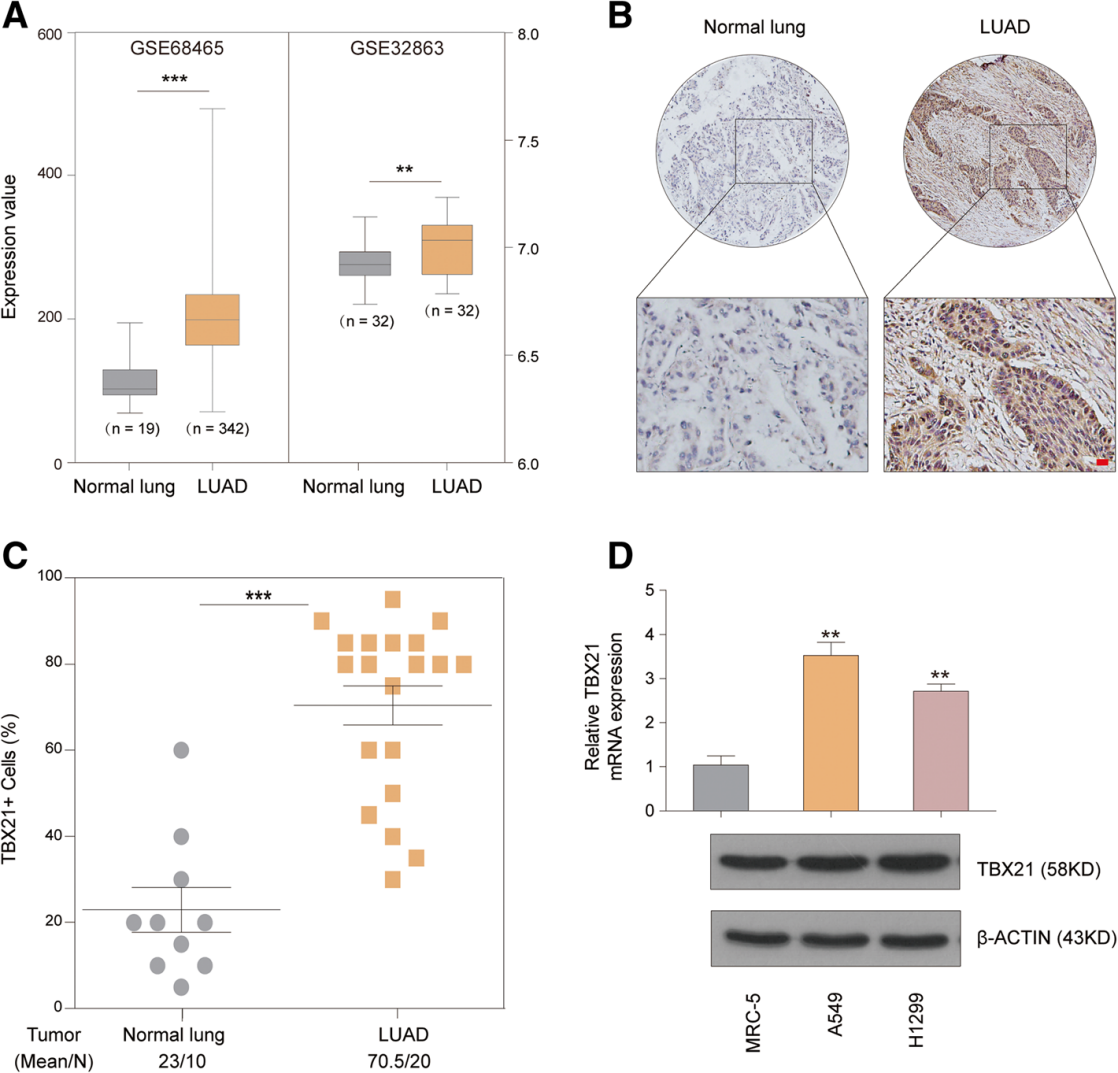
Validation of expression status of TBX21 between normal samples and LUAD. **a** mRNA expression of TBX21 is significantly upregulated in LUAD compared with normal lung in GSE32863 and GSE68465 (***P < 0.0001, **P < 0.01, *t*-test method). **b**, **c** Immunohistochemistry assay shows protein expression of TBX21 is significantly upregulated in LUAD compared with normal lung tissue (***P < 0.001, *t*-test method). **d** Expression of TBX21 is significantly downregulated in normal cell line (MRC-5) compared with tumor cell lines (A549 and H1299) examined by quantitative RT-PCR method (bar plot, top panel) and immunoblot analysis (bottom panels). Error bars represent SEM (**P < 0.01, *t*-test method). LUAD lung adenocarcinoma

To confirm the high expression of TBX21 in LUAD, the TBX21 gene was firstly evaluated between adjacent normal and tumor tissues using a microarray assay from 64 samples in GSE32863 and 361 samples in GSE68465. We found that it was significantly upregulated in LUAD tissues compared with normal tissues (*P* < 0.01; Fig. 2a). Then the protein expression of TBX21 was determined using an immunohistochemistry assay in LUAD and normal lung sections from 15 samples (10 LUAD tissues and 5 adjacent normal tissues). The results showed that the expression of TBX21 was significantly higher in LUAD samples than in normal samples (*P* < 0.0001; Fig. 2b, c), which was also in line with the mRNA expression profile earlier. These results demonstrated that an increased expression of TBX21 might be associated with the presence of LUAD.

To determine whether TBX21 was upregulated in tumor cell lines, the expression of TBX21 was measured with quantitative RT-PCR and immunoblot analysis between one normal cell line (MRC-5) and two LUAD cell lines (A549 and H1299), respectively. As a result, we discovered that not only mRNA expression but also protein expression of TBX21 was increased in LUAD cell lines compared with the normal cell line (Fig. 2d). Therefore, these studies suggested that these cell lines could be utilized to validate the activated role of TBX21 in LUAD genesis.

### Validation of stemness-related role of TBX21 in LUAD maintenance

**Fig. 3:**
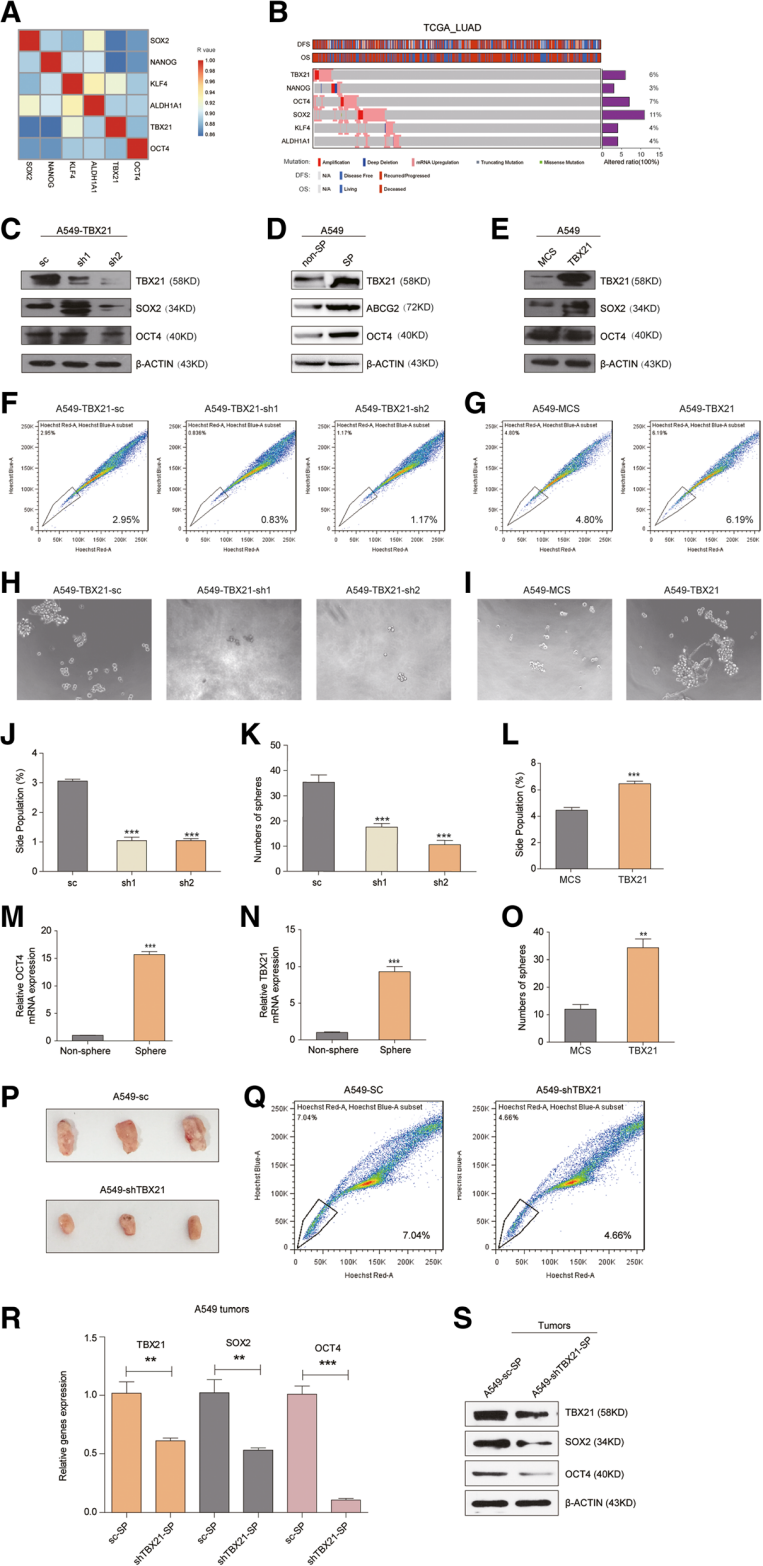
TBX21 associated with cancer stemness as well as CSC biomarkers. **a** Heatmap shows significantly high correlation between TBX21 and CSC biomarkers (SOX2, NANOG, KLF4, ALDH1A1 and OCT4), Spearman’s *r* ≥ 0.85. **b** Genetic alterations analysis of TBX21 and five CSC biomarkers in LUAD patients (*n* = 520) with DFS or OS status from TCGA including amplification, deep deletion, mRNA upregulation, truncating mutation and missense mutation. Synthetically altered ratios 6%, 3%, 7%, 11%, 4% and 4% for TBX21, NANOG, OCT4, SOX2, KLF4 and ALDH1A1 in LUAD patients, respectively. **c** Western blot analysis of SOX2 and OCT4 expression with TBX21 knockdown in A549 cells. β-ACTIN is loading control. **d** Western blot analysis of TBX21 expression in non-SP or SP cells. β-ACTIN is loading control. **e** Western blot analysis of SOX2 and OCT4 expression with TXB21 ectopic expression. β-ACTIN is loading control. **f** Side population assay to detect SP cell proportion with silencing of TBX21. **g** Side population assay to detect SP cell proportion with TXB21 ectopic expression. **h** Representative images of tumor spheres with TBX21 deficiency. **i** Representative images of tumor spheres with TBX21 overexpression. **j** Statistical results of percentage of SP cells with TXB21 knockdown. **k** Statistical results of tumor spheres with TXB21 knockdown. **l** Statistical results of percentage of SP cells with TXB21 overexpression. **m** qPCR analysis of OCT4 expression at mRNA level in sphere or nonsphere cells. **n** qPCR analysis of TBX21 expression at mRNA level in sphere or nonsphere cells. **o** Statistical results of tumor spheres with TXB21 overexpression. **p** Representative images of tumor burden 12 days after delivery of A549-shTBX21 or A549-sc cells into NOD/SCID mice. **q** Side population assay to detect SP cell proportion in suspension derived from A549-shTBX21 or A549-sc tumors. **r** Statistical results of percentage of SP cells between A549-shTBX21 and A549-sc tumors. **s** Western blot analysis of TXB21, SOX2 and OCT4 expression between A549-shTBX21 and A549-sc tumors. β-ACTIN is loading control. ***P < 0.0001, **P < 0.01, *t*-test method. LUAD lung adenocarcinoma, TCGA The Cancer Genome Atlas, DFS disease-free survival, OS overall survival, N/A not available, sc control shRNA, SP side population, MCS multiple cloning site

Previous studies reported that CSC biomarkers such as NANOG [21, 22], OCT4 [21, 23], SOX2 [21, 24], KLF4 [21, 25] and ALDH1A1 [21, 26] were associated with a poor prognosis in several cancers. The correlation analysis indicated that the average expression of the five cancer stemness biomarkers was tightly associated with TBX21 in the integrated GEO dataset (*r* ≥ 0.850, *P* < 0.05; Fig. 3a). Further, we evaluated the expression of these five CSC biomarkers and TBX21 by analyzing the RNA sequence of tumors and the matched non-neoplastic tissue from 520 LUAD patients with disease-free survival status or overall survival status in the TCGA dataset. The result summarized the overall landscape of the significantly mutated genes in LUAD, and the patients with mutation (amplification, deep deletion, missense mutation and mRNA upregulation) altered in 154 (30%) of 520 patients (Fig. 3b). Also, we discovered that mRNA upregulation was the dominating mutation form among the five CSC biomarkers and TBX21 (Fig. 3b). We also discovered that the total mean mutation rates of SOX2 and OCT4 (11% and 7%, respectively) were higher than the other three CSC biomarkers (3% for NANOG, 4% for KLF4 and 4% for ALDH1A1, respectively) in these patients, which was close to the TBX21 mutation rates (6%; Fig. 3b). So, we could infer that TBX21 might play a positive role in LUAD maintenance of cancer stemness.

To validate the activated function of TBX21 in mediating cancer stemness in LUAD, the expression of TBX21 was knocked down in A549 cells by stable expression of the control shRNA (sc) or two shRNAs for TBX21. Consistently, the expression of cancer stemness biomarkers, including SOX2 and OCT4, was decreased following the downregulation of TBX21 expression in western blot analyses (Fig. 3c). Then, flow cytometry of TBX21-transfected A549 cells was performed to identify side populations and the result showed that the proportion of side-population (SP) cells was significantly depressed by the loss of TBX21 (*P* < 0.0001; Fig. 3f, j). As an important feature of cancer stemness, self-renewal of LUAD cells was measured in the sphere formation experiment of A549 cells with sc or shRNAs for TBX21. A549-TBX21-sc cells readily grew spheres in serum-free medium and the presence of TBX21 markedly increased the number and size of spheres compared to that of A549-TBX21-shRNAs (Fig. 3h, k). Moreover, TBX21 expression was measured in SP cells sorted with flow cytometry and evaluated by the protein expression of ABCG2 and OCT4 with western blot assay (Fig. 3d), and in cultured spheres evaluated by mRNA expression of OCT4 with RT-PCR analysis (Fig. 3m). In line with expression of ABCG2 and OCT4, expression of TBX21 was significantly higher in SP cells (*P* < 0.0001; Fig. 3d) and tumor spheres (*P* < 0.0001; Fig. 3n) compared with non-SP cells or nonspheres. In addition, the TBX21 gene was reconstituted into A549 cells and then increased the protein expression of the stemness markers, including SOX2 and OCT4 (Fig. 3e). In agreement, ectopic expression of TBX21 raised remarkably the proportion of SP cells (*P* < 0.0001; Fig. 3g, l) and enhanced ability dramatically in the tumor sphere formation of A549 cells (*P* < 0.0001; Fig. 3i, o). Taken together, these data indicated that TBX21 promoted the cancer stemness of LUAD cells and played a positive role in maintaining LUAD cell development.

To further identify the role of TBX21 in regulating cancer stemness in vivo, we performed tumor xenograft studies in NOD/SCID mice injected with either A549-shTBX21 or A549-sc cells. Similarly, the result showed a marked reduction in tumor growth in the A549-shTBX21 group versus the A549-sc control (Fig. 3p). Furthermore, flow cytometry was conducted in the A549-shTBX21 or A549-sc tumors to identify side populations and the result showed that SP cells were significantly depressed by the loss of TBX21 (*P* < 0.0001; Fig. 3q). Then, mRNA and protein expression of TBX21, SOX2 and OCT4 were measured in both SP cells with RT-PCR analysis and western blot method, respectively. As a result, we discovered that both the mRNA and protein expression of TBX21, SOX2 and OCT4 in SP cells derived from A549-shTBX21 tumor were significantly declined compared with those from A549-sc tumor (*P* < 0.01; Fig. 3r, s). In line with the result in vitro, these data indicated once again that TBX21 could improve the cancer stemness of LUAD cells and positively maintain LUAD development.

### IL-4 signaling is indispensable for TBX21 to define cancer stemness

**Fig. 4:**
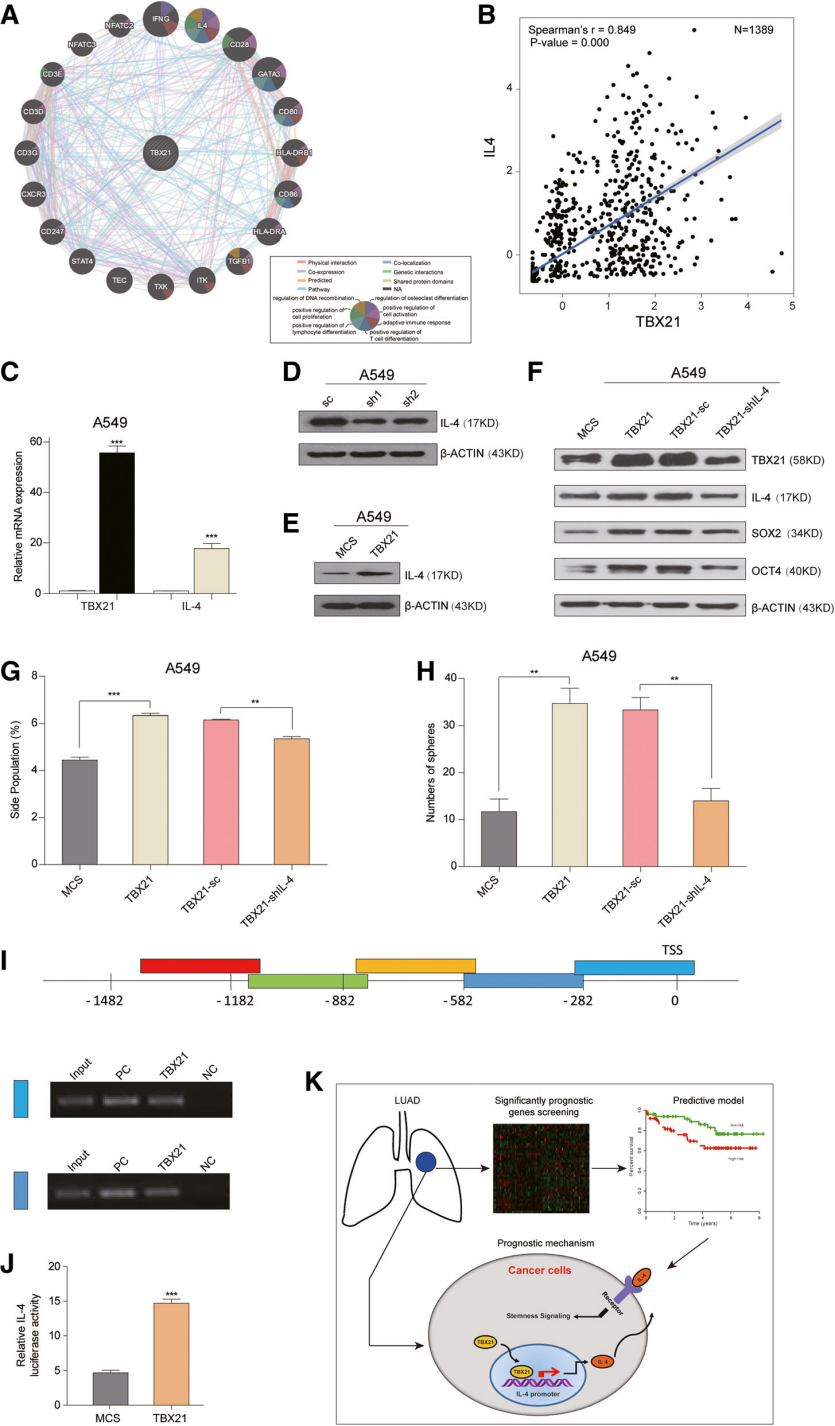
IL-4 is targeted by TBX21 in A549 cells to maintain cancer stemness. **a** Functional enrichment map for RNA-based expression revealing TBX21 modulated networks. Nodes (circles) with distinct color subparts represent different function enriched in gene sets from DAVID database. Nodes including distinct color subparts have significant overlap with TBX21 predicted targets, and each node connected with TBX21 belongs to the same cluster (MCL cluster algorithm). Distinct color lines between nodes correspond to different interactions among these significant genes (Wilcoxon test *P* < 0.05). **b** Correlation of TBX21 and IL-4 mRNA levels (normalized) in integrated dataset. Spearman’s correlation and *P* value indicated. **c** qPCR analysis of TBX21 and IL-4 expression at mRNA level. **d** Western blot analysis of IL-4 expression with TXB21 knockdown in A549 cells. β-ACTIN is loading control. **e** Western blot analysis of IL-4 expression with TXB21 ectopic expression. β-ACTIN is loading control. **f** Western blot analysis showed IL-4 deficiency rescued SOX2 and OCT4 expression in A549-TBX21 cells. β-ACTIN is loading control. **g** Statistical results of percentage of SP cells with IL-4 knockdown in A549-TBX21 cells. **h** Statistical results of tumor spheres with IL-4 knockdown in A549-TBX21 cells. **i** CHIP analysis of TBX21-binding sites on IL-4 gene promoter. **j** Dual-luciferase assay to detect IL-4 promoter activity after TBX21 overexpression. **k** Summary of prognostic model associated with LUAD. Schematic includes two lungs with LUAD, microarray screening, predictive model and mechanism investigation. Blue cell in left lung represents LUAD lesion. Heatmap indicates significant genes correlated with prognosis. ***P < 0.0001, **P < 0.01, *t*-test method. Ellipse demonstrates signaling pathway of TBX21–IL-4 in cancer cells. N/A not available, IL interleukin, sc control shRNA, TSS transcription start site, LUAD lung adenocarcinoma, MCS multiple cloning site, PC positive control, NC negative control

To identify potential biological processes and pathways involving the TBX21 gene, functional enrichment analysis was performed by GO terms and KEGG pathways between TBX21 and the other protein-coding genes (PCGs). Next, Spearman’s correlation coefficient was calculated between TBX21 and the PCGs using paired expression profiles, and the top 20 positively or negatively correlated PCGs were chosen with at least one kind of relationship such as physical interaction, coexpression, predicted, pathway, colocalization, genetic interaction and shared protein domains (Fig. 4a). The result revealed that IL-4 associated with TBX21 clustered the most GO functional clusters (including DNA recombination, cell proliferation and activation, immune cell and osteoclast differentiation, and adaptive immune response; Fig. 4a). The average expression of IL-4 was tightly correlated with TBX21 in 1389 patients with LUAD from the six GEO datasets (Spearman’s *r* = 0.849, *P* = 0.000; Fig. 4b). As previous studies proved that IL-4 with high expression value promoted cancer stemness [27] and predicted the poor prognosis [28] in cancers, the results indicated that cancer stemness might depend on the interaction between TBX21 and IL-4 in LUAD cells.

To validate the correlation between TBX21 and IL-4 in cancer stemness regulation, the expression of both genes was detected with RT-PCR. As shown in Fig. 4c, mRNA expression of IL-4 was significantly upregulated following the reconstitution of TBX21 expression in A549 cells. Accordingly, the IL-4 expression was detected both in TBX21 deficiency and reconstitution systems with western blot analysis. Obviously, the protein expression of IL-4 was decreased with TBX21 deficiency (Fig. 4d) and increased with TBX21 reconstitution (Fig. 4e). These results suggested that IL-4 might be targeted by TBX21 to mediate cancer stemness.

To prove this conclusion, IL-4 gene was knocked down in the TBX21 reconstitution systems in A549 cells. The result showed that expression of SOX2 and OCT4 was correspondingly reduced accompanied by the loss of IL-4 (Fig. 4f). Furthermore, the SP cells were evaluated with flow cytometry, and the result showed that IL-4 gene deficiency significantly abolished the SP cell increase induced by the TBX21 gene (Fig. 4g). In concordance with the SP, loss of IL-4 expression was discovered to abolish the sphere formation induced by TBX21 (Fig. 4h). Some studies revealed that TBX21 works as a transcription factor and regulates many target genes [14, 29, 30]. The Chip assay was then performed to investigate whether TBX21 could regulate IL-4 gene transcription. The result verified that TBX21 binds to IL-4 gene promoter with a region of approximately 0–600 bp upstream of the transcription start site (TSS) (Fig. 4i). To complement the Chip-PCR assay, IL-4 promoter-derived luciferase reporter construct and TBX21-overexpressed vector were cotransfected into A549 cells. The luciferase assay confirmed that the overexpression of TBX21 really heightened IL-4 promoter activity (Fig. 4j). Taken together, a schematic model correlated with survival of LUAD patients was displayed focusing on the relationship between the TBX21–IL-4 signaling pathway and cancer stemness. TBX21 works as a transcription factor targeting the promoter region of IL-4 which promotes IL-4 expression and secretion. The secreted IL-4 bound to its receptor on the cancer cell membrane further induced downstream signaling activation to mediate cancer stemness (Fig. 4k). All of the presented results prove that cancer stemness maintenance was governed by the TBX21–IL-4 pathway in LUAD cells.

## Discussion

LUAD is the most common kind of NSCLC [31]. LUAD patients were treated with surgical resection for early stage disease and adjuvant chemotherapy for stage IB or II. Although oncologists usually made the treatment decisions for adjuvant chemotherapy according to the traditional clinical factors such as age, gender, tumor stage, tumor size and so on, LUAD patients still suffered from the high risk of cancer recurrence. A recent study focusing on the molecular mechanisms of LUAD showed that LUAD was a heterogeneous cancer including diverse morphologic and molecular features [32]. The response to adjuvant chemotherapy has been proven to correlate with the heterogeneous molecular features [33]. So, it is urgent to develop some new molecular biomarkers to further stratify LUAD patients for identifying cancers with a high-risk value who will benefit from the adjuvant chemotherapy and patients with a low-risk value who will be able to avoid overtreatment.

In the past time, some studies of LUAD have reported the ability to generate expression signatures or a single gene to group subjects according to their survival outcomes [34–38]. However, little has been reported for the inflammatory gene associated with cancer stemness to predict survival of patients with LUAD in a large dataset. The published gene TBX21 shows little overlap with the others as a significant predictor of outcome in LUAD. Thus, there is a strong possibility that this TBX21 gene could be used for diagnosis and prognosis prediction with large samples and adequacy of the different microarray platforms, contributing dramatically to the rational results.

In this study, we collected different datasets to generate a significant gene TBX21 to construct the prognostic model that could have potential clinical implementation. Significant emphasis was placed on the validation of this model by using a similar cutoff method to reduce technical variability in the other five GEO datasets. In the univariate and multivariate analysis, the significant factors represented the biology of the LUAD and associated clinical data. The prognostic power of TBX21 was analyzed with the log-rank test method and a significant result was obtained between patients with high-risk score and those with low-risk score. The OS rates of patients with high-risk score were significantly lower than those with low-risk score at 3 and 5 years, respectively. The median survival year was remarkably shorter for patients with high-risk score compared to those with low-risk score. A realistic assessment of the performance of this predictive model was executed in these datasets, and the HRs between high-risk and low-risk groups for all the datasets were more than 1.40. Furthermore, data stratification was performed among the three significant clinical factors (stage, age or gender) according to the risk level of TBX21 in LUAD patients from all GEO datasets. The rational results suggest that the prognostic model is independent of the other clinical factors. Especially in line with the research hypothesis, the predictive model of TBX21 could differentiate patients with poor survival and favorable survival within the same stage, age or gender stratum, which indicated the potential application of the prognostic model in predicting survival of patients with LUAD.

Importantly, it remains unknown whether the model has predictive power in LUAD patients from different clinical centers in China as this TBX21 predictive model was derived from the GEO datasets. Another limitation of our study is that the test set is not large enough to allow for a sensible assessment of generalizability for this predictive model. Datasets from other clinical departments and other countries are still necessary to validate its generalization ability. The validity of this gene model should be further confirmed in the prospective cohorts. Finally, our predictive model could potentially be applied to other NSCLC cancer types such as lung squamous cell carcinoma (LSCC). Our preliminary work in LSCC has shown equivalent results with those in LUAD (Additional file 3: Figure S2), but much more data for LSCC is needed to research their presence and clinical relevance in future.

Although more and more biomarkers were identified to predict the prognosis of LUAD during the past years [39–41], functional study of biomarkers is still limited. Using the LUAD cell line A549, we discovered that TBX21 could promote sphere-forming capacity concomitant with upregulated expression of stemness biomarkers SOX2 and OCT4, suggesting that TBX21 increases the self-renewal of lung CSCs. In addition, TBX21 promotes the proportion of SP cells to display augmented drug resistance, another property associated with CSCs. Importantly, TBX21 induces cancer stemness biomarker (SOX2 and OCT4) alteration in LUAD cells and the mice model which is consistent with the acquisition of CSC maintenance. Overall, our studies provide the first evidence that TBX21 promotes cancer stemness which may contribute to LUAD growth, metastasis and recurrence.

Differentiation of CD4^+^ Th1 and Th2 αβ cells is tightly crossregulated, so that development of one subset is inhibited by cytokines produced by the other [29, 42]. Considerable progress has been made in understanding the molecular mechanisms of this crossregulation. TBX21, an identified Th1-specific transcription factor selectively expressed in Th1 cells, plays a central role in Th1 development by activating Th1 genetic programs and repressing Th2 cytokine synthesis [30]. GATA3, in contrast, is a Th2-specific transcription factor selectively expressed in Th2 cells. It plays a major role in specifying the Th2 phenotype by promotion of Th2 cytokine secretion and inhibition of IFN-γ production through repression of IL-12 signaling [29]. To our surprise, in our system our results showed that TBX21 and GATA3 have the same mRNA expression trends (Additional file 4: Figure S3). Moreover, our functional data underscore that TBX21 promoted IL-4 expression and IL-4 signaling is indispensable for TBX21-mediated cancer cell stemness. It is suggested that the reverse roles of genes may have the same contribution in tumor development.

## Conclusions

In summary, we identified a novel model to predict survival in patients with LUAD by integrating currently available datasets. Based on the TBX21 expression value, we developed a risk score model dividing patients into a high-risk group and a low-risk group with significantly different prognosis. The robustness of this predictive model was successfully validated between the training dataset and the other five independent datasets for validation. Importantly, the prognostic power of this new model was derived from cancer stemness governed by the TBX21–IL-4 signaling pathway and was independent of the other clinical factors. Our research highlighted the potential roles of TBX21 as a new predictive biomarker and therapeutic target for LUAD patients.

## Additional files

Additional file 1: **Table S1.** Presenting 1027 inflammation-related genes measured in this study. Loza et al*.* [20] identified associations between novel variants in inflammation-related genes and several common diseases, especially for some cancers. Genetic studies also provided evidence that some cancers were associated with inflammatory pathways which impact the risk of cancer initiation, progression and severity. (XLSX 17 kb)

Additional file 2: **Figure S1.** Showing stratification analysis of the TBX21 gene for stage, age and gender in integrated dataset including training dataset and five validation datasets. Kaplan–Meier survival curves between high-risk group and low-risk group for (**A**) stage I patients, (**B**) stage ≥ II patients, (**C**) young patients, (**D**) older patients, (**E**) female patients and (**F**) male patients. (TIFF 1760 kb)

Additional file 3: **Figure S2.** Showing prognostic model of TBX21 could be applied to patients with LSCC. (**A**) Heatmap shows genetic alterations analysis of TBX21 and five CSC biomarkers in LSCC patients (*n* = 504) with DFS or OS status from TCGA including amplification, deep deletion, mRNA upregulation, truncating mutation and missense mutation. (**B**) Synthetically altered ratios 5%, 10%, 6%, 60%, 6% and 6% for TBX21, NANOG, OCT4, SOX2, KLF4 and ALDH1A1 in LSCC patients, respectively. (**C**) Kaplan–Meier curves for overall survival between high-risk group and low-risk group defined by our predictive model based on TBX21 expression values in TCGA dataset. (**D**) DFS analysis of LSCC patients when stratified into high-risk group and low-risk group by TBX21 prognostic model in TCGA dataset. (TIFF 1239 kb)

Additional file 4: **Figure S3.** Showing TBX21, IL-4 and GATA3 gene expression in A549 cells. qPCR showed that mRNA expression of IL-4 and GATA3 was significantly upregulated with reconstitution of TBX21 expression in A549 cells (*P* < 0.001, *t*-test method). (TIFF 201 kb)

## Abbreviations

CI: Confidence interval
CSC: Cancer stem cell
GEO: Gene Expression Omnibus
HR: Hazard ratio
IFN-γ: Interferon gamma
IRG: Inflammation-related gene
LSCC: Lung squamous cell carcinoma
LUAD: Lung adenocarcinoma
NSCLC: Nonsmall cell lung cancer
PCG: Protein-coding gene
TCGA: The Cancer Genome Atlas
TSS: Transcription start site
