# Supplementary figures and images for: TBX21 predicts prognosis of patients and drives cancer stem cell maintenance via the TBX21–IL-4 pathway in lung adenocarcinoma
Source: Stem Cell Res Ther. 2018 Apr 3;9:89. doi: 10.1186/s13287-018-0820-6 (PMC5883886; doi:10.1186/s13287-018-0820-6)

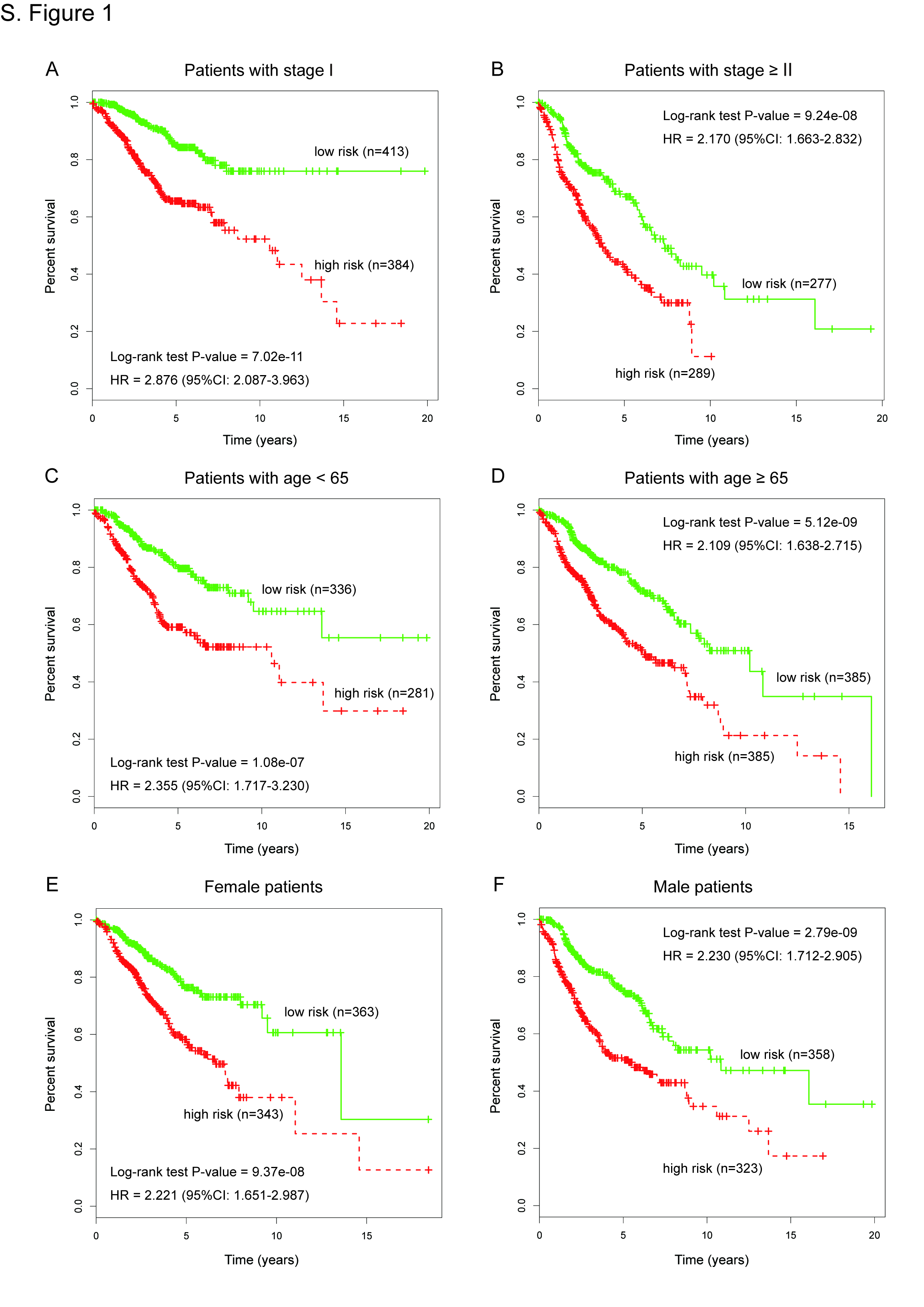

Supplement: Supplementary file 2 — Figure S1. Showing stratification analysis of the TBX21 gene for stage, age and gender in integrated dataset including training dataset and five validation datasets. Kaplan–Meier survival curves between high-risk group and low-risk group for (A) stage I patients, (B) stage ≥ II patients, (C) young patients, (D) older patients, (E) female patients and (F) male patients. (TIFF 1760 kb) [file 13287_2018_820_MOESM2_ESM.tif]

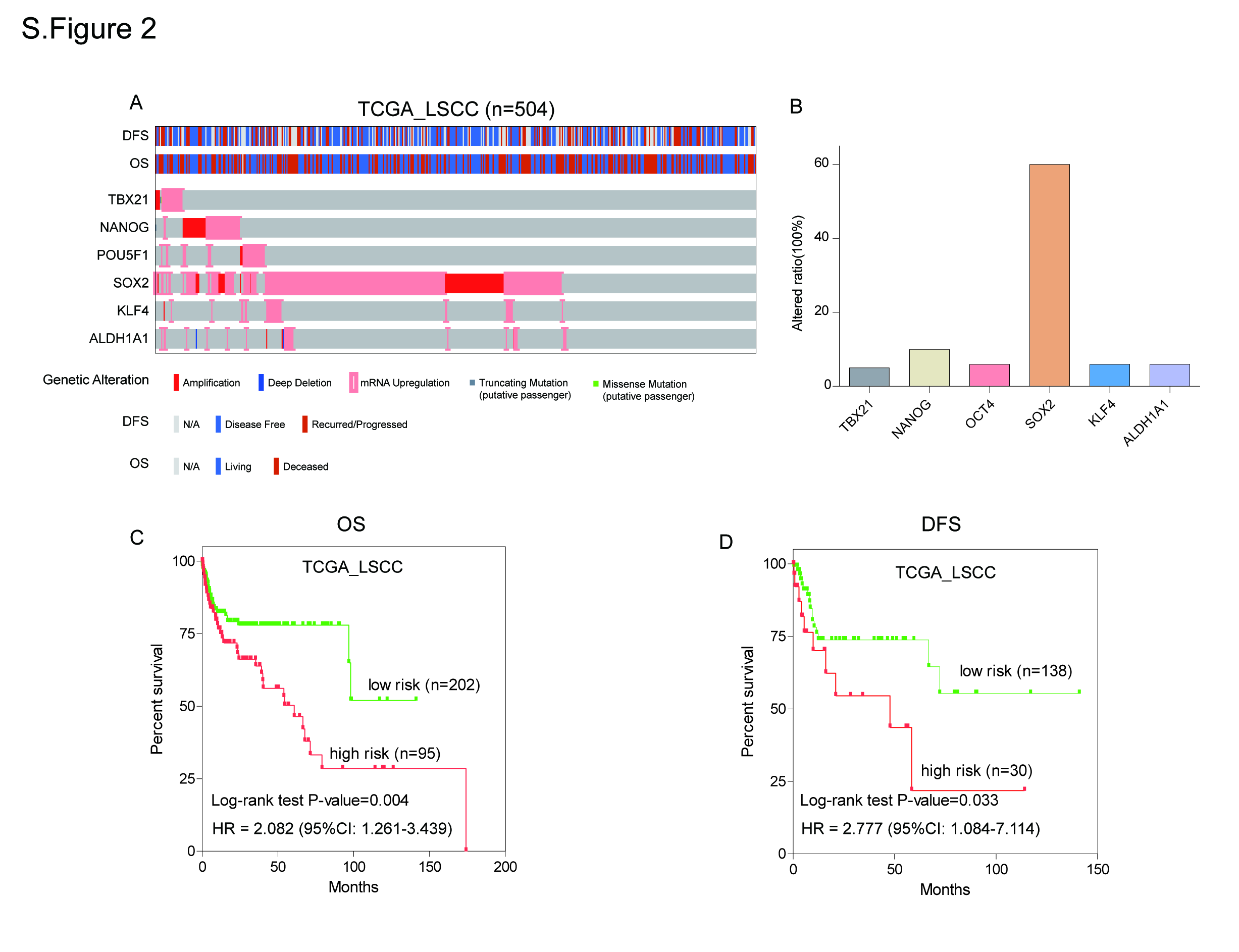

Supplement: Supplementary file 3 — Figure S2. Showing prognostic model of TBX21 could be applied to patients with LSCC. (A) Heatmap shows genetic alterations analysis of TBX21 and five CSC biomarkers in LSCC patients (n = 504) with DFS or OS status from TCGA including amplification, deep deletion, mRNA upregulation, truncating mutation and missense mutation. (B) Synthetically altered ratios 5%, 10%, 6%, 60%, 6% and 6% for TBX21, NANOG, OCT4, SOX2, KLF4 and ALDH1A1 in LSCC patients, respectively. (C) Kaplan–Meier curves for overall survival between high-risk group and low-risk group defined by our predictive model based on TBX21 expression values in TCGA dataset. (D) DFS analysis of LSCC patients when stratified into high-risk group and low-risk group by TBX21 prognostic model in TCGA dataset. (TIFF 1239 kb) [file 13287_2018_820_MOESM3_ESM.tif]

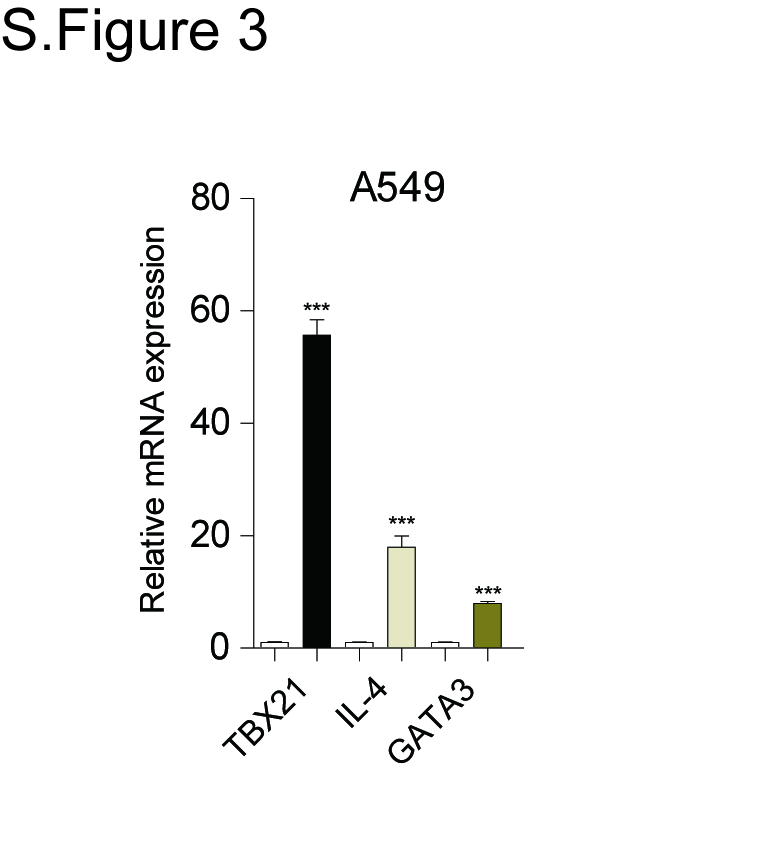

Supplement: Supplementary file 4 — Figure S3. Showing TBX21, IL-4 and GATA3 gene expression in A549 cells. qPCR showed that mRNA expression of IL-4 and GATA3 was significantly upregulated with reconstitution of TBX21 expression in A549 cells (P < 0.001, t-test method). (TIFF 201 kb) [file 13287_2018_820_MOESM4_ESM.tif]
